# Brain Tumour Segmentation Using Convolutional Neural Network with Tensor Flow

**DOI:** 10.31557/APJCP.2019.20.7.2095

**Published:** 2019

**Authors:** M Malathi, P Sinthia

**Affiliations:** *Saveetha Engineering College,Chennai, India. *

**Keywords:** Brain tumour- magnetic resonance imaging- convolutional neural network- segmentation

## Abstract

**Introduction:**

The determination of tumour extent is a major challenging task in brain tumour planning and quantitative evaluation. Magnetic Resonance Imaging (MRI) is one of the non-invasive technique has emanated as a front- line diagnostic tool for brain tumour without ionizing radiation.

**Objective:**

Among brain tumours, gliomas are the most common aggressive, leading to a very short life expectancy in their highest grade. In the clinical practice manual segmentation is a time consuming task and their performance is highly depended on the operator’s experience.

**Methods:**

This paper proposes fully automatic segmentation of brain tumour using convolutional neural network. Further, it uses high grade gilomas brain image from BRATS 2015 database. The suggested work accomplishes brain tumour segmentation using tensor flow, in which the anaconda frameworks are used to implement high level mathematical functions. The survival rates of patients are improved by early diagnosis of brain tumour.

**Results:**

Hence, the research work segments brain tumour into four classes like edema, non-enhancing tumour, enhancing tumour and necrotic tumour. Brain tumour segmentation needs to separate healthy tissues from tumour regions such as advancing tumour, necrotic core and surrounding edema. This is an essential step in diagnosis and treatment planning, both of which need to take place quickly in case of a malignancy in order to maximize the likelihood of successful treatment.

## Introduction

Dimililera et al., (2016) discussed Brain tumour is the abnormal growth of cells within the brain. There are two main types of tumours like benign and malignant tumours. Generally, tumour is classified into primary and secondary tumour. Primary tumour starts within the brain and secondary tumour will spread to the other parts of the body. There are many medical imaging methods available like X-ray, CT (Computed Tomography) and MRI (Magnetic Resonance Imaging). The research article uses MRI brain images, because of its high resolution and good quality of an image. After capturing MRI brain image, it is necessary to separate the tumour region from the MRI brain image. Accurate segmentation of medical images helps the radiologist for radiotherapy planning. The most common type of tumours is such as astrocytoma. oligodondroglima and glioblastama. The presented work mainly segments glioma types of brain tumour. The astrocytoma is the most common type of glioma which is formed by star shaped cells. Segmentation of tumour from brain images is critical, because of its complex structure of the brain tissues so, it is compulsory to provide further diagnosis. Further, it is difficult to perform accurate delineation in radiotherapy. Since, it is compulsory to avoid injury to the sites of language, motor sensory function during therapy. The manual segmentation of brain tumour is labour sensitive and the segmentation results based on the operators experience and their subjective decision making. Hence there is a need for fully automatic, objective and reproducible segmentation methods. There are many challenges and fully automatic algorithms for brain tumour segmentation, because of its high variability of brain tumour size, shape, regularity, location and their heterogeneous appearance. Rémi et al., (2018) discussed the proposed work classifies the high grade gliomas. Generally, astrocytomas are the most common type of glioma found in both adults and children. Astrocyotomas are classified into low grade gliomas and high grade gliomas. The work uses fully automatic convolutional neural network. The convolutional neural network is implemented in python programming. The anaconda is one of the frameworks for machine learning concept, in which neural network tool for training of BRATS database is implemented using tensor flow. By using this, the accuracy of segmentation is improved and it has the features to process the larger dataset. The performance of the segmentation of MRI brain tumour is compared with the ground truth images of BRATS database 2015. The dice co-efficient is the parameter to define the accuracy of automatic segmentation.

*Relevant work and our contributions*

The various brain tumour segmentation techniques have been discussed as follows:

Dong et al., (2017) proposed noninvasive magnetic resonance techniques as a diagnostic tool for brain tumour to identify brain tumour without ionizing radiation. Manual segmentation of the 3D MRI volumes needs larger time, and the performance is mainly based on the operators experience. Hence, the author recommended a u-net based deep convolution network. This segmentation is implemented on BRATS 2015 datasets, which contain 220 high grade glioma brain tumour and 54 low grade tumour cases. The performance of our proposed method was compared to the manual delineated ground truth U-net based deep neural network provides the superior results for the core tumour regions. 

Haveri et al., (2017) illustrated a brain tumour segmentation using deep neural networks to glioblastomas (both low and high grades) MRI image. This kind of brain tumour appears anywhere in the brain and also it has any shape, size and contrast. The article utilizes the convolutional neural network as a machine learning algorithm. It exploits both local and global features for tumour segmentation. The author uses BRATS dataset for research work.

Isin et al., (2016) discussed that segmentation of brain tumour is one of the challenging tasks in medical field. The lifetime of the patient is improved by early diagnosis of brain tumour. Manual segmentation of brain tumour for large amount of data is a time consuming process. Hence there is a need for automatic segmentation. Now a days the automatic segmentation uses deep learning techniques for segmentation. It provides efficient segmentation for a large amount of MRI based image data. The article reviewed the state of the art methods of deep learning. The conventional automatic segmentation methods need prior knowledge into probabilistic maps or selecting highly representative features for classifiers, which is challenging task. But, the convolutional neural network method automatically learns the corresponding complex features for both healthy brain tissues and tumour tissues from multimodal MRI brain images.

Pereira et al., (2016) described gliomas is one of the aggressive types of brain tumour which leads to the short lifetime with their highest grade. Since, the MRI techniques produce large amount of data, manual segmentation needs reasonable time. The automatic brain tumour is one of the challenging tasks due to the large, spatial and structural variability among brain. The author suggested a new segmentation method based on a convolutional neural network with small 3×3 kernals. The small kernel helps the deep architecture to avoid over fitting by assigning the fewer number of weights in the network. And also using intensity normalization as a preprocessing along with convolutional neural network provides effective segmentation. Further, the proposed work is implemented BRATS 2013 data base.

Hussian et al., (2017) suggested a segmentation algorithm to detect the gliomas based brain tumour. It uses deep convolutional neural network algorithm to locate the tumour which has an irregular shape. Hence, the survival rate of the patient is increased with accurate segmentation of brain tumour. The problem of overfitting is removed by introducing max-out and drop out layers in the patch processing. The proposed algorithm also uses a preprocessing method to remove the unwanted noise and post processing help to remove small false positive using morphological operators. The research work uses BRATS 2013 dataset for segmentation.

Wang et al., (2017) recommended that convolutional neural network provides the state of the art performance for automatic medical image segmentation. But, it does not provide robust results for clinical use. It has the limitation to the lack of generalizability of previously unseen object classes. The problem is rectified by novel deep learning based interactive segmentation framework by relating CNN into bounding box and scribble based segmentation pipeline. The proposed method makes the CNN model adaptive to a specific test image, which may be unsupervised or supervised.

Cui et al., (2018) developed a novel automatic segmentation based on cascaded deep learning convolutional neural network. It has two sub networks; tumour localization network (TLN) and a intra tumour classification network (ITCN). The tumour region from the MRI brain slice is separated using tumour localization network and ITCN helps to label the defined tumour region into multiple sub-regions. The work was performed on multimodal brain tumour segmentation (BRATS, 2015) dataset, which had 220 high grade glioma (HGG) and 54 low grade glioma (LGG) cases. The evaluation can be performed by dice coefficient, positive predictive value (PPV) and sensitivity. 

Khawaldeh et al., (2018) offered a widespread machine learning technique for medical image classification and segmentation. The approach uses conv net for classifying brain medical images into healthy and unhealthy brain images. The implemented method classifies the brain tumour into low grades and high grades. It uses alex krizhousky network deep learning architecture to classify the MRI brain tumour. The tumour classification is performed on the whole image rather than pixels.

Chinmayi et al., (2017) conferred a method for MRI brain tumour segmentation and classification using Bhaltacharya co-efficient. The unwanted skull portions were removed using anisotropic diffusion filter. Further, it uses a fast bounding box algorithm to extract the tumour area. It uses deep learning CNN to train the MRI brain tumour image. Finally, the results of the proposed method compared in terms of accuracy, similarity index, PSNR and MSE. The results will help the radiologist to identify the size and position of a tumour.

Konstantinos et al., (2017) developed a segmentation of brain lesion which is a challenging task, was performed using the 3-D convolutional neural network. The dual pathway architecture was used to extract the local and larger contextual information, which operates an input image at multiple scales. The false positives which were removed by using 3D fully connected conditional random field. The segmentation process was used to separate lesion on multichannel MRI with traumatic brain injuries, brain tumours and ischaemic stroke. The 3-D CNN is an effective method, which provides good segmentation without increasing the computational cost and the number of training parameters.

In modern years, several methods have been implemented to automatically segment MRI brain tumours. These methods can be basically divided into two categories respectively hand- crafted features and classifier method based on traditional learning such as support vector machine (SVM) and random forest. The second method is based on fully automatic methods based on deep learning using the Convolutional Neural Network (CNN). The first category method use manually separated features and it is given as input to classifiers. After determination of hand crafted features. The classifiers do not modify the features during training. But in second category the features, specifications can be adapted to do specific task of training data. Currently deep neural network CNNs mostly used in the computer vision community.

## Maerials and Methods

The research article uses convolutional neural network for MRI brain tumour segmentation using tensor flow. Normally, the segmentation is performed using various tools like MATLAB, LABVIEW etc. The research article uses tensor flow based MRI brain tumour segmentation in order to improve segmentation accuracy, speed and sensitivity. Segmentation can be performed on BRATS MRI brain images and results are compared in terms of dice co-efficient.

*Python Based Convolutional Neural Network*

Many researchers use MAT LAB to implement the segmentation process. Our research work utilizes the python programming to implement the segmentation of MRI brain tumour. The features are listed below in order to choose python programming to implement the research work

1. Python code is more compact and readable than MATLAB

2. The python data structure is superior to MATLAB

3. It is an open source and also provides more graphic packages and data sets

**Figure 1:**
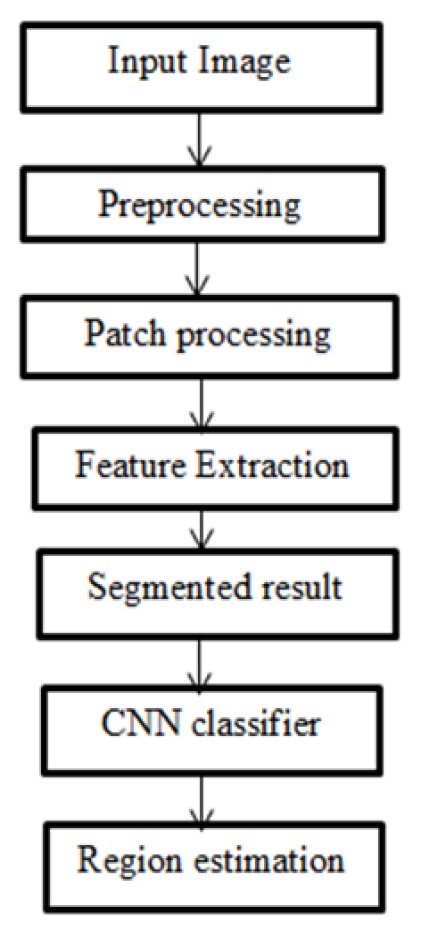
Predictive Model of Proposed of Work

Hence, the proposed work utilizes python programming instead of MAT LAB. There are some additional python packages used during the implementation process of our research work through python. The flow diagram of the proposed work as shown in Figure 1.

*MRI Brain data acquisitions*

**Figure 2:**
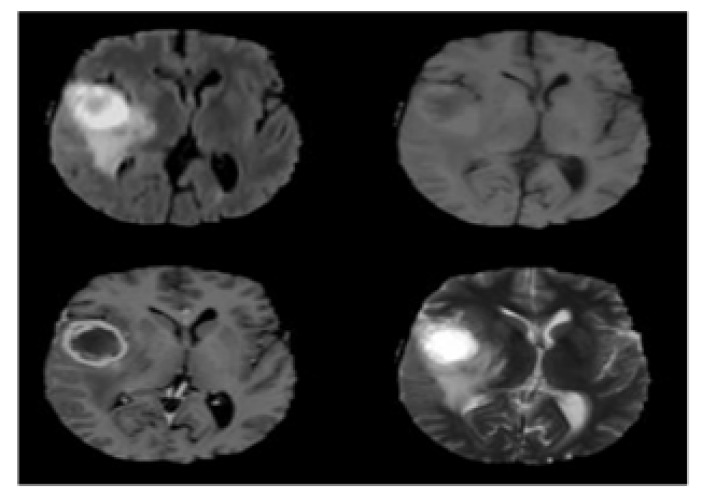
Pulse Sequence Images of Brain Tumour

The recommended method was tested and implemented on the BRATS 2015 dataset, which had 220 high grade glioma (HGG) and 54 (LGG) low grade glioma patient scanned images. Each patient in the BRATS 2015 dataset multimodal MRI was available and also four scanning sequences were implemented for every patient using T1 weighted (T1), T1 weighted imaging with gadolinium enhancing contrast (T1C), T2 weighted and FLAIR. The article uses the HGG image of BRATS 2015. Pulse sequence images of brain tumour as shown in Figure 2.

*Preprocessing*

**Figure 3:**
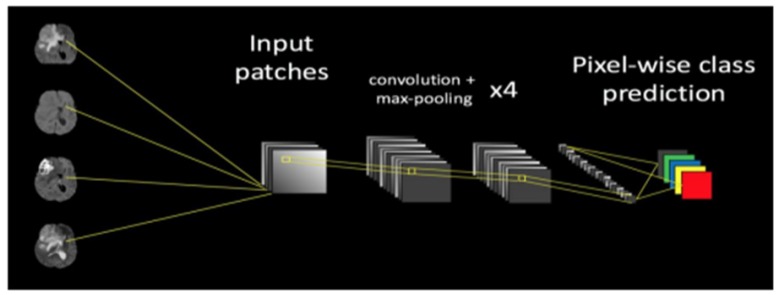
The Architecture of Convolutional Neural Network

The main challenging task is removing artifacts produced by inhomogeneity in a magnetic field or small movements created by the patient during scanning. Many time bias is present in the scanning results, which affect the segmentation results, particularly in the computer n based models. Chmelika et al., (2018) demonstrated work uses n41TK bias correction for the T1 and T1C images in the data set. The n4T1K bias correction removes the intensity gradient on each scanning images. Additionally, noise reduction is also performed by median filter in order to standardize the pixel intensities. Hence, noise reduction and bias correction helps to improve the data processing and provides the better segmentation, multiple radio frequency pulse sequences can be used to provide the different types of tissue. In BRATS data base there are four different sequences available for each image like fluid attenuated recovery (FLAIR), T1, T1 contrasted and T2. The chemical and physiological characteristics can be obtained from these pulse sequences, which result in contrast between the individual classes. The architecture for the proposed work as shown in Figure 3.

*Neural network*

For image recognition, neural network is one of the powerful tools to perform segmentation. MRI is one of the most commonly used imaging techniques to capture MRI brain images. Automatic segmentation is a challenging task because of its large spatial and structural variability. Hence, the proposed system implements the automatic segmentation method based on CNN exploring small 3×3 kernals. The small size kernals help to design deeper architecture by using fewer number of weights in the network.

*CNN algorithm*

Raphael Prevost et al., (2018) discussed CNN algorithm performs the voxel-wise classification problem. The tumour or lesion portion is separated from the background by calculating the probability of each image voxel belonging to the target is known. The CNN network has four sections input and convolution sections. The input layer processes the input image in order to produce the designed image patches. The convolution section process the designed image patches, in which multilayer convolutional filters operates and output feature maps. Further, the fully connected layer that groups all feature maps. The classification section estimates a prediction score to classify the every image voxel and provides a segmentation map.

**Figure 4:**
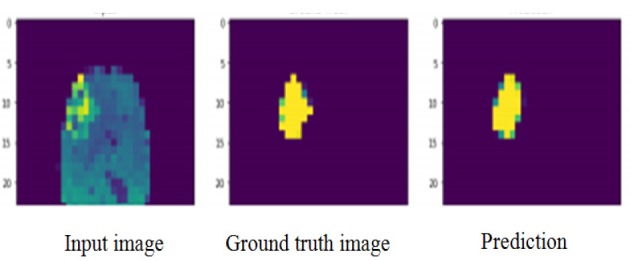
Segmented Image of Brain Tumour

**Figure 5:**
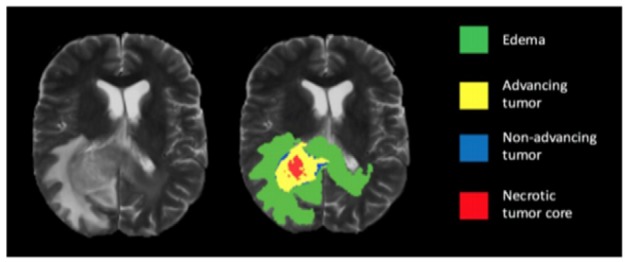
Result of Brain Tumour Segmentation

**Figure 6:**
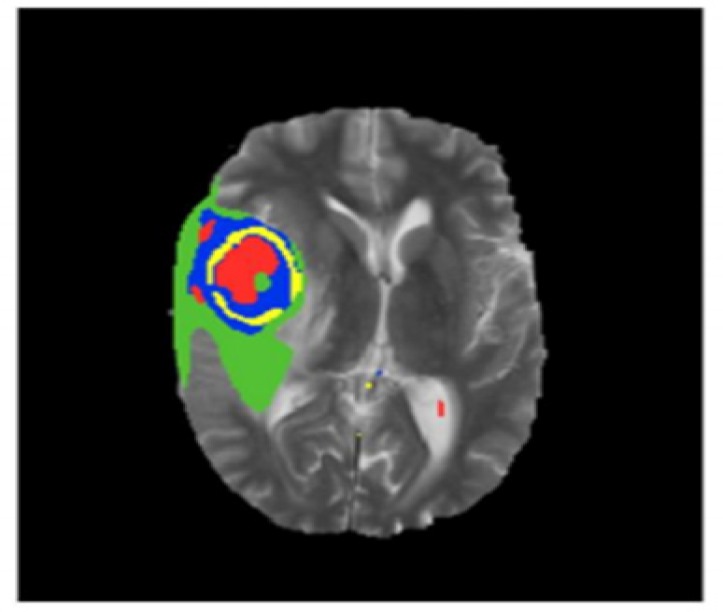
Brain Tumour Segmentation with Different Labels

**Table 1:** Parameters for Measurement of Various Ground Truth Brain Images

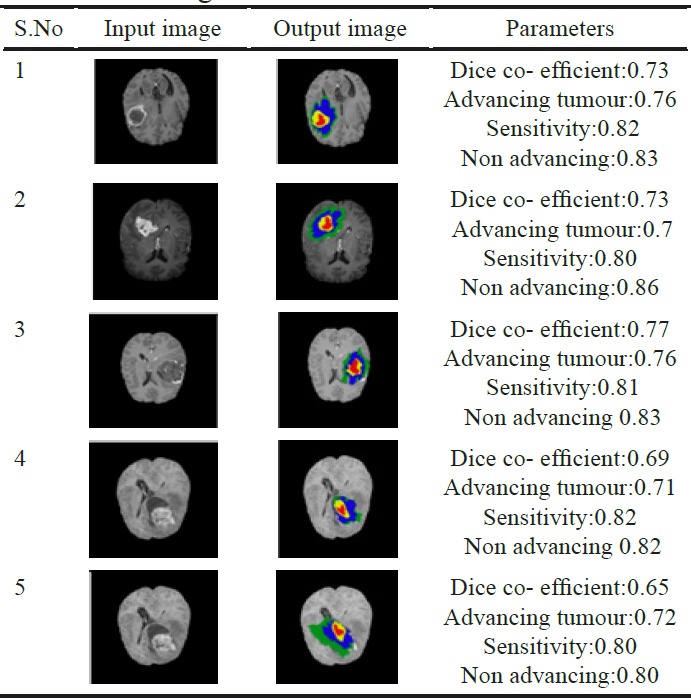

*Input section*

The input section generates the image patches for the remaining of the network. Chena et al., (2017) implemented The method performs the classification based on voxel-wise, where each voxel is classified based on the linear and nonlinear relationship between the focal voxel’s intensity and its neighbours. The input 3D image size is large; hence, calculation of linear and nonlinear relationship between all voxels in the complete image is complex. Hence, the entire image is divided into smaller patches in order to find the relationship within a particular region instead of the entire image. It reduces the computational time and also memory space. It separates both local and global patches as input for the convolution section. For every extraction process, the central voxel is chosen randomly and extracted concentric local and global image patches. The neighbouring voxel around the central voxel provides the local information and global patch covers larger region and provides global information. In order to reduce the computation burden produced by the larger global patch we used down sampling of all global patches. The down sampling process is also called as pooling. 

*Convolution section*

There are multiple layers in the convolution section, which help to sequentially identify the features using convolution operations. The captured features are low level features like edges and corner relationship between neighbouring voxels. The feature maps are the output of the convolutional layer. The complete convolution region consists of three different sub paths. The path 1 and 2 utilize the same local patch with various filter sizes to internment various neighbourhood patterns, at the same time subpath 3 uses downsampled global patch to provide global features. The convolutional layer calculates the output of the neurons that are connected to either local or global regions in the input. The convolution is the process of performing dot product between their inputs and their receptive field to which they are connected to in the input volume. For our research work 

L- be the depth of the convolution filter stack

a^1^ (1Ɛ [1,L]) represents the number of feature maps in the l^st^ layer and 

F_i_^l^ (iƐ [1,α^l^] ) represents the i^th^ feature map of the l^st ^layer F_i_^l^ is calculated by

$F_{i}^{l}=g(\sum_{j=1}\propto^{l-1}F_{j}^{i-1}*w_{j^{1}}^{l}+b_{i}^{i}$

Where g(.) is the PReLU function. The PRELU is a neuron activation function defined by 

g(x)= x, x >0

ax x≤0

a is the network parameter

W_j1_^l^ is the filter connecting the j^th^ feature map in the l-1^th^ layer and b_i_^l^ is the bias of the artificial neuron model

ReLU is the rectified linear units.

It is one of the most popularly used activation functions. It is represented as 

R(x)= max( 0, X) ie 

If X < 0 R(x)=0

If X>=0, R(x)=X

Most of the machine learning techniques refers to this function because of its simplicity and also it avoids vanishing gradient problem. But, it should be used only within the hidden layer of neural network model. For example, output layers, softmax function will be used to calculate the probabilities for the classes. During the training phase, some gradients can be fragile and die. Hence, it needs a weight update, further, it never activates on any data point again. It can be avoided by another modification called leaky ReLU. It provides small slope to keep updates alive.

*Fully connected region*

The special information is preserved in the fully connection section by fusing of all of the feature maps produced by the convolution section. The operation of fully connected section is similar to the convolution section in which each co-efficient node performs like a convolution filter with the size (1,1,1)

*Classification section*

The classification section helps to provide probability for each voxel. Most of the Convolutional neural network uses softmax function in order to map the feature maps into categorical probabilities.

$J_{p}\left(\mathrm{\emptyset .}I_{s}.C_{s}\right)=-\frac{1}{B∙w}\sum_{S=1}^{B}\sum_{V=1}^{V}\log(P_{C_{s}^{v}}(x^{v}))$

Where *x*^v^ and *C*_s_^v^ are the v^th^ target voxel’s position and ground truth, 

$V\in \left[1\mathrm{.V}\right]∙V=f_{1}^{L}\times f_{2}^{L}\times f_{3}^{L}$

Where (*f*_1_^L^, *f*_2_^L^*,* *f*_3_^L^) is the L^th^ layer feature map size. S ϵ [1, B] where the B is the size of a batch, *P*_Cs_^v^ is the output of the softmax function. The parameter* θ* is denoted as filter co-efficients and bias. It can be found by training through the stochastic gradient descent method. SGD is one of the stochastic approximations of the gradient descent optimization method in order to minimize an objective function iteratively. For every iteration, the method estimates the gradient from a subset of labeled dataset and also it helps to increase the speed of training in a huge training set.

*Training of network*

The training model is created using Keras. The 4 layer sequential model was trained on selected 33× 33 patches of MRI images in order to classify the center pixel. Every input has 4 channels, among four, one channel helps for each imaging sequence and the net can learn the relative pixel intensities of each given class.

*Validation of data set*

The proposed method uses BRATS database for evaluating the brain tumour segmentation methods. BRATS provides each patient’s T_1_ weighted MRI with gadolinium contrast (T_1c_) and T_2_ weighted fluid attenuated inversion recovery (FLAIR) images. This kind of data set compares our automatic delineation method with other algorithms.

## Results

The BRATS data base provides different target structures edema (label 1), advancing tumour. 

(Label 2), non-advancing tumour (label 3), necrotic tumour core (label 4) and background.

The proposed work uses either colour, gray scale or intensity images with a default size of 220×220. The automatic brain tumour detection of a patient consists of two important stages, namely, image segmentation and edge detection. The next important step is adding path from the image data set file to the system directory. It can be implemented by an anaconda frame work to allocate data sets from computer memory. It can be performed either manually or by programming using the python OS inbuilt operation.

The main objective of segmentation is to cluster pixels into image region and it helps in identifying a region of interest ie., to locate tumour and other abnormalities. The pixels segmented portion is compared with normal brain image in the jupytor note book. This kind of comparison helps to locate abnormal parts of brain tumour patient. The next step is edge detection. The work uses cannon edge detection in order to classify the brain tumour portion of the segmented image. The white portion shown is tumour. In the resultant, red represents the necrotic core of the tumour, yellow represents the advancing tumour, blue represents non-advancing tumour and green represents the swelling part of the tumour called edema. The results of the segmentation process are shown in Figure 4, 5 and 6.

## Discussion

*Dice similarity coefficient*

In mathematical notation, dice score is the number of false positives divided by the number of positives added with the number of false positives.

*Experiments and performance evaluation*

Five-fold cross validation method is used for evaluation of HGG and LGG data respectively. For every patient, four subnormal regions are validated. It is represented by 

a) The complete tumour region (It includes all the four intra tumoural classes label 1,2,3 and 4)

b) The core tumour region (It excludes edema region, (label1), includes label (2, 3, 4)

c) The advancing tumour (only label 2)

For every tumoural region, the performance of segmentation has been evaluated using the dice similarity co-efficient and the sensitivity. The DSC measures the overlap between the manual delineated brain tumour regions and the segmentation results of our fully automatic method that is 

$\mathrm{DSC}=\frac{2\mathrm{TP}}{\mathrm{FP}+2\mathrm{TP}+\mathrm{FN}}$

TP- True positive

FP –False positive

FN-false negative

Additionally, sensitivity is also used to measure the number of TP and FN that is

$\mathrm{Sensivity}=\frac{\mathrm{TP}}{\mathrm{TP}+\mathrm{FN}}$

The table provides the Dice co-efficient value for different ground truth brain images.

In conclusion, the segmentation of brain tumour plays an important role in diagnostic procedures. The accurate segmentation helps in clinical diagnostic, but also helps to increase the lifetime of the patient. In this paper a segmentation of brain tumour implemented using CNN architecture. The algorithm relates both local and global features, because it helps to perform the segmentation accurately. The training and testing speed is increased by using max pooling, max out and drop out complement the learning process. The speed is increased by reducing the features in the fully connected layer. Reducing of parameters also causes the reduction of overfitting. The result shows that the implemented method helps in detection of enhancing tumour as well as specifying tumour to the actual tumour region only.

## Conflicts of interest

None .
